# Self-Care and Self-Management Among Adolescent T2DM Patients: A Review

**DOI:** 10.3389/fendo.2018.00489

**Published:** 2018-10-18

**Authors:** Jafrin Jahan Eva, Yaman Walid Kassab, Chin Fen Neoh, Long Chiau Ming, Yuet Yen Wong, Mohammed Abdul Hameed, Yet Hoi Hong, Md Moklesur Rahman Sarker

**Affiliations:** ^1^Faculty of Pharmacy, Universiti Teknologi MARA, Puncak Alam, Malaysia; ^2^Faculty of Pharmacy, Cyberjaya University College of Medical Sciences, Cyberjaya, Malaysia; ^3^Collaborative Drug Discovery Research Group, Pharmaceutical and Life Sciences Community of Research, Universiti Teknologi MARA, Shah Alam, Malaysia; ^4^School of Pharmacy, KPJ Healthcare University College, Negeri Sembilan, Malaysia; ^5^Pharmacy, School of Medicine, University of Tasmania, Hobart, TAS, Australia; ^6^Faculty of Pharmacy, Universiti Teknologi MARA, Bertam, Malaysia; ^7^Department of Physiology, Faculty of Medicine, University of Malaya, Kuala Lumpur, Malaysia; ^8^Department of Pharmacy, State University of Bangladesh, Dhaka, Bangladesh

**Keywords:** diabetes, type 2 diabetes mellitus, adolescent diabetes, self-care, self-management

## Abstract

Uncontrolled hyperglycaemia can lead to macro- and microvascular complications. Adolescents with T2DM develop similar complications as in adults, including cardiovascular disease, stroke, myocardial infarction, renal insufficiency, and chronic renal failure. Although regular medical follow-up is essential to avoid long-term complications, patients with diabetes mellitus need to perform holistic self-care activities such as opting for a healthy diet, physical activity, self-monitoring, and proper medication. To the best of our knowledge, only a limited number of studies have focused on self-care activities and self-management, including self-care practices, supportive networks, and self-care education programs in adolescent with T2DM. Some of the studies focused on the appreciation of self-care in adolescents with T2DM. This review aimed to analyse self-care and self-management among adolescents with T2DM, and discuss the impact of self-care and self-management on glycaemic control. The difficulties faced by adolescents in self-managing their disease are also highlighted. Such information is essential for healthcare providers in promoting self-care practices among adolescents with T2DM. A thorough search of the literature was performed using three databases: Medline, Google Scholar, and Scopus. The articles focused on self-care and self-management of adolescents patients with T2DM aged between 12 and 19 years old were included. Findings from this review reveal that healthy food adaptation, adequate physical activity, proper medication practices, and regular glucose monitoring are the most common self-care practices. Parental involvement and clinician encouragement also contribute toward the practice of self-care and self-management among the adolescents with T2DM. In conclusion, independent self-management regimens and supportive networks for appropriate administration are vital factors to enhance clinical outcomes of adolescents with T2DM.

## Introduction

Diabetes mellitus (DM) is a metabolic disorder of multiple etiologies that is characterized by chronic hyperglycaemia due to impairments in insulin release, insulin actions or both. In 2004, the “SEARCH for diabetes in youth” study from the United States reported the prevalence of diabetes in youth between the age of 10 and 19 is 1 out of 357 young people (1). The prevalence of Type 2 diabetes mellitus (T2DM) in adolescents is estimated as 0.19 cases per 1000 non-Hispanic white youth and 1.74 cases per 1000 American Indian youth (2). Recently, studies have reported that T2DM is a worldwide health issue, and its prevalence has increased steadily globally (3).

The World Health Organization (WHO) estimates that the mortality rate associated with DM will double by 2030 (4). The associated complications include stroke, myocardial infarction, kidney damage, early death and eye damage (5). Alteration of lifestyle, including healthy diet, daily exercise and monitoring of blood-glucose levels may delay the progession of T2DM (6). American Association of Diabetes Educators (AADE) suggests that a person healthy lifestyle, diet, monitoring and maintenance of glucose level, and medication adherence should be strictly followed by diabetes patients (7). American Diabetes Association (ADA) advocates that weight loss, monitoring the intake of carbohydrate, and fiber, restriction of cholesterol, saturated fat, trans fat, sodium as the integral part of DM treatment (8). In addition, it is suggested that patients required additional approaches to address individual, family, and social practice to improve self-management (9).

Self-care is defined as the care that incorporates any deliberate moves to look after physical, mental and emotional health. The patient decision and behavior that they engage in any chronic disease that affects their well being is the best characteristics of self-management (10). Self-care practices involve a variety of areas that includes food, exercise, medicine, emotion, sleep, and medical care (11).

Given the importance of self-care and self-management of diabetes is imperative for adolescent DM patient, it is necessary to review the self-care practices among these patients.

## Methodology

The review focused on the impact of self-care and self-management among adolescents with T2DM aged between 12 and 19 years old. The literature search was performed using three electronic databases (i.e., Medline, Google Scholar, and Scopus). Additional studies were identified from the bibliography of the articles. The following search terms were used: “T2DM,” “adolescent diabetes,” “self-care,” and “self-management.” This review only included the studies with an interventional approach that focused on the self-care practices and self-management among the adolescent T2DM patients aged between 12 and 19. Conference abstracts, editorial letters, review papers and non-English literature were excluded. The scopes of self-care and self-management among adolescent patients with T2DM were divided into three sections: self-care practices, efficiency of self-care support system, and assessment of self-care.

### Self-care practices

Diabetes self-care is a transformative process of improvement of information in the social surroundings by figuring out how to cope with the complex environment (12, 13). It is essential to have reliable and substantial measures for self-care and self-management of diabetes (14, 15).

Diabetes self-care behavior refers to the practices embraced by individuals with or at the risk of DM in order to manage their disease effectively by themselves (11). Seven major diabetic self-care practices include healthy diet, physically dynamic, glucose monitoring, proper medication, excellent problem-solving attitudes, sound adapting abilities, and risk-reduction (11). All these seven self-care practices are associated with good glycaemic control, problem reduction and improvement in quality of life (16, 17). The type of activities of self-care and self-management for DM are presented in Table 1 (18–20).

**Table 1:** Activities of self-care and self-management of diabetes.

**Category**	**Activities**
General (18)	Diabetes education: Organize training sessions (routinely investigated, strengthened and diagnosed) for better understanding of the patient. Diet and routine activities of life: Prepare a healthy eating routine, weight reduction activities if the individual is overweight and regular physical activities. Maximize glucose control while minimizing unfriendly effects of treatment, for example, hypoglycemia. Reduce the risk of diabetes complications, including the early recognition and management of hypertension. Medication treatment is required to adjust lipid levels and consideration of antiplatelet therapy with aspirin. Monitor the early medication for difficulties associated with diabetes, including cardiovascular disease, feet issues, and neuropathy.
Medication (19)	Ensure proper, customized drug training is accessible for family or parental figures, and that healthcare experts overseeing medications are suitably qualified and equipped. Consider the medication load and lessen polypharmacy. Use the lowest compelling dosage, increase measurements gradually and screen the impacts, including adverse effects. The administration of medicine times must match with meal times if the person is on insulin or sulfonylureas to reduce the risk of hypoglycaemia.
Meal & Nutrition (20)	The nutrition plan ought to be individualized and consider individual food preferences, eating schedules, religion and culture, and physical and intellectual health status. The meal plan ought to incorporate a variety of foods to ensure sufficient amounts of essential vitamins, minerals, protein, and fiber are consumed. Higher protein and healthy food consumption might be expected to enhance the nutritional and functional status in fragile older individuals with diabetes.
Physical activity & Exercise (20)	Exercise is one of the most overlooked types of self-care. Physical activity and proper exercise should be considered in relation to the drug regimen, particularly glucose-lowering agents associated with an expanded risk of hypoglycaemia. To encourage a low-force home-based workout program to enhance physical execution and keep up a good ADL and mobility. The most secure effective support for exercise that individuals can undertake is from family members and guardians.

Obesity is the main problem for most adolescent patients with T2DM (21, 22). Dietary intervention is a vital element for weight reduction in the management and treatment of obesity. A few studies have focused on obesity management in children (23, 24). Only a limited studies focused on the treatment and management of adolescent patients with T2DM that have incorporated dietary intervention in combination with exercise and behavioral strategies (25–29). The findings from these studies showed negative results in regard to the impact of diet on treatment outcomes; however, the independent effects of dietary changes was not evaluated. A recent study compared long-term reduced glycemic load diets with standard reduced-fat diet among obese adolescent patients. It showed that reduced glycemic load diet can be a good substitute to a conventional reduced-fat diet for lowering diabetic complications in obese adolescents with T2DM (30).

Self-management of exercise and physical activity is an integral part of controlling diabetes and assisting with the movement of the skeletal muscle. The major aims of exercise are to facilitate the regulation of blood glucose, improve insulin action, fat and protein metabolism, avoid diabetic complications, and enhance life expectance (31). An article aims to equip the primary care providers with the current standard of care of T2DM management in youth emphasizes an management of goal of normalizing glycemia and HbA1c, enhancing diabetes self-management capacities, increasing exercise, reducing weight, and improving nutrition (32).

Adequate physical activity is associated with a lower HbA1c provided that it is coupled with dietary guidance (33). Youth should understand the importance of routine exercise, which helps them to burn calories, lose excess weight, and control glucose levels (33). Moreover, the combination of dietary changes and regular exercise helps to maintain normal weight and enhance weight loss in overweight/obese people. The United States Department of Health and Human Services recommends exercise of at least 30 to 60 min, most days of the week, for overweight patients (34). Proper management of diet is one of the barriers for diabetic adolescent patients. For example, adolescents struggle to stay away from standard adolescent favorites, including fast food, fries, and sweets. The study also reported that mother's involvement in maintaining a healthy diet showed positive results in term of diabetes control and stress levels (35). Adaptation of healthy lifestyle behavior may have a significant effect on the diabetes status of a patient. The implementation of healthy lifestyle behavior among adolescent patients means better mental health and good glycemic status (36, 37).

### Efficiency of self-care support system

Self-care support can be described as a group of people, including health-care professionals, family and friends, providing an individual with practical or emotional support (38). To encourage patients to perform self-care activities, it is necessary to manage diabetes and to adapt to this devastating situation (39). There is a paucity of studies investigating the impact of social involvement and self-care management of diabetes in young people with T2DM. A study demonstrated that young people with diabetes encounter the same formative directions as healthy adolescents in physical, enthusiastic, social, and behavioral development, and thus family and peer group acceptance and support might be imperative for disease management (40–42).

The capacity of young people with T2DM to deal with their condition is affected by a scope of elements together with social, natural, and individual factors (38). It is recommended that lifestyle modification for the management of T2DM in adolescents should be guided and monitored by family members, as better clinical outcomes can be achieved among those youths who involve both parents and themselves in diabetes management (43). Studies by La Greca et al. are among the earliest to indicate that overemphasis on urging youngsters to accomplish freedom in diabetes self-care may lead to worse clinical outcomes. Hence, utilizing a child's age alone as a manual to determine suitable self-care autonomy should be discouraged (44, 45).

An adolescent can face lots of difficulties in learning new things as their behavior following the diagnosis of diabetes may change remarkably. During this stage, parental support and involvement are vital. Also, these young people normally expect full support from their family (46). Psychological control harms children as it interrupts their self intellectual development; while, behavioral control benefits children because it gives them desirable guidance, without essentially inhibiting their individuation (47, 48). Two studies demonstrated that regular monitoring and continued support from parents are essential, whereas the irregular involvement of parents in adolescent diabetes care can result in poor outcomes for diabetes management (49, 50). Research has demonstrated that rebellious approaches to cope with diabetes are harder and associated with inferior psychosocial adjustment, and it may be that these adolescents have already negotiated a level of attachment that is comfortable for them, so family involvement does not interfere with their quality of life (50).

It is perceived that social support from family and friends can decrease the stress that young people with T2DM encounter. Peer and parental support can indeed encourage young people with T2DM to perform self-care practices and alteration, adapt to a diabetes diagnosis, and engage in self-care practices. A study involving 74 adolescent diabetes patients was carried out to assess the support that adolescent patients received from their friends during treatment. The impact of support from friends was not significant in the prolonged treatment but had a great impact on the adherence with blood-glucose monitoring (51). A similar study was conducted to assess and analyze the effect of the support given by the family and companions for youngsters in diabetes care. The study concluded that families pay more attention than friends in three different types of support (insulin infusions, blood-glucose checking, and meals). However, in an emotional affair, adolescents get more support from friends rather than family (52). The adolescent may not always feel comfortable discussing their disease with everyone. Healthcare professionals could play an important role in supporting them to make friendly confessions about their condition with those close to them. Healthcare professionals could help young people in figuring out a way to discuss their disease management or ask their peers about the ideal approaches to assist them in managing their disease (53).

Moreover, this review highlights that the collaborative care is an important criterion of self-management for adolescent diabetes patients. If all the supportive groups play their role, then it is easy for adolescents to manage their diabetes properly.

### Assessment of self-care

The term self-management is frequently baffling as there is no generally acknowledged definition, and it is utilized to convey different ideas, for example, the guidance of self-care and self-management, patient activities, and self-management education (38). Self-management education enhances control of T2DM, particularly when conveyed as short intercessions, enabling the patient to recollect and have a better blend of information (54). The conventional educational forms of care that include instructing patients to enhance the awareness of health status provide a path to the present forms that focus on the behavioral and self-care advances aim to equip patients with the attitudes and strategies to advance and alter their behavior (55).

Self-management education is a community-oriented and continuing process expected to encourage the advancement of behaviors, knowledge, and abilities that are required for fruitful self-management of diabetes (56). A multidisciplinary team is essential for the education program which involves educational supporters from hospitals and clinics, and the direct involvement of healthcare professionals. The process of the education program ought to comply with the standards and terms stated by the National Standards for Diabetes Self-management Education, which aims to support and assist diabetes educatiors in providing good quality education and self-management support (56).

The American Association of Clinical Endocrinologists has recognized that Diabetes Self-Management Education (DSME) remains as a crucial feature of care for diabetes people. In addition, DSME serves as an avenue for acquisition of knowledge, skills, abilities, and collaboration with other people, which are essential for engaging self-management of diabetes (57). DSME programs help individuals to adapt to the psychological and physical needs of the disease, specifically the remarkable financial, social, and cultural conditions. The principal objective of DSME is to enable patients to take control of their own condition by enhancing their insight and attitudes, so that, they can make knowledgeable decisions for self-guided behavior, changing their regular lives and eventually moderating the danger of complications (58). Definite metabolic control and quality of life as well as the avoidance of complications are the ultimate aims specified by diabetes self-management education (59).

Knowledge of and information about the successful management and treatment of adult diabetes patients allow adjustments to be made in youth's management of diabetes. The treatment and management guidance of adult patients needs to be translated and adapted by child patients. Though these guidance are easily translatable to older adolescents, physicians are often hesitant regarding how to treat and manage young children and adolescents with T2DM (60). Through knowledge and education, individuals with DM can figure out how to make life decisions, and can discuss more with their clinicians to accomplish ideal glycemic control (61). A study examined the impacts of a self-care education program on T2DM patients demonstrated that the program leads to an improvement in state of mind and behavior, and fewer complexities, and thus leads to an improved mental and physical quality of life. (62). Several authors have discussed that diabetes self-management education is provided to control the disease including monitoring of emergencies such as hypoglycemia and hyperglycemia. Indeed, several studies found that diabetes self-management education improves HbA_1C_ and patient compliance (63, 64). A diabetes education program is vital in glycemic control, as psychological support brings better clinical outcomes and emotional improvement, and controls the hazard of continuing complications (64–66).

Among the primary barriers of managing youth and children with T2DM are inadequate scientific support about treatment, patient adherence, and deficiency in knowledge about recent recommendations (67, 68). Consequently, various ways have been recommended for self-management of diabetes mellitus among adolescents. These provide a coherent picture of daily activities and care that adolescent patients with T2DM adapt effectively (69). To accomplish this goal, further interventional work is required to positively establish the most efficient management alternative in this population. The previously published studies in this setting are summarized in Table 2.

**Table 2:** Studies of self-care and self-management of adolescent patients with diabetes.

**References**	**Year**	**Diabetes type**	**Objectives**	**Population**
Krenke (49)	1998	Type 1 & 2	To determine the atmosphere of families with diabetes adolescents and healthy adolescents.	195
Pinhas-Hamiel et al. (22)	1999	Type 2	To recognize the involvement of the family in the management criteria including behavioral, physical, and social characteristics of T2DM adolescents patients.	42
Ebbeling et al. (30)	2003	Type 2	To assess the outcome of glycemic load and reduced fat diet among obese diabetes patients.	16
Lipton et al. (42)	2003	Type 1 & 2	To identify the outcomes of self-management of diabetes and social issues in children of a specific ethnic group in Chicago.	288
Berry et al. (32)	2006	Type 2	To highlight the overall management of T2DM in youngsters.	
Mulvaney et al. (68)	2008	Type 2	To construct out the insight of self-management and possible barriers among adolescents with T2DM mellitus.	24
Rothman et al. (28)	2008	Type 2	To look at self-management activities of adolescents T2DM patients and the glycaemic effect.	139
Polikandrioti and Dokoutsidou (31)	2009	Type 2	Discuss the function of dietary management and exercise in T2DM.	
Auslander et al. (35)	2010	Type 2	The study focused on the African American adolescent patients and their mother and aimed to recognize the psychosocial assets.	20
Brouwer et al. (53)	2012	Type 2	To identify the Type 2 adolescent knowledge with distinguished peer supports.	8
Berkowitz et al. (37)	2017	Type 2	To determine the association between healthy lifestyle behavior and the treatment option by changing glycemic control and obesity.	234
McGavock et al. (36)	2018	Type 2	To determine the association between healthy lifestyle behavior and mental health in T2DM adolescent patients.	162

## Future research and directions

Further research is essential to get a more reliable conclusion concerning the appropriate self-care practices and self-management of adolescent patients with T2DM. Most studies were conducted on self-care practices and self-management in adult patients with T2DM. There is a number of quality studies of self-care practices with type 1 adolescent patients, but only a small number have included type 2 adolescent patients. Nevertheless, adult diabetes management approaches are successful for imparting knowledge and understanding, and are adaptable for adolescents (60).

Although the management process of adolescents is almost same as the adults, healthcare providers are usually uncertain about how to guide and develop the knowledge and understanding of the most appropriate methods for proper management guideline for adolescents with T2DM. There are very limited experimental trials, and most of the treatment and management recommendations are referred from adults; therefore, the current guidelines for management for adolescents with T2DM may not be fully evidence-based. Successful outcomes have been noticed for both Type 1 and T2DM in youth and adolescent patients through a supportive team. Given the recognized importance of social support in encouraging diabetes self-care behaviors, family and care-givers could lessen the burden of T2DM by providing extra attention to the patients' need (41, 53).

Research highlights the necessities of self-care and self-management for those who have a delayed determination of diabetes, a period where intercessions can lead the most significant advantages for long-term education opportunities and management. Early concerns and active management are imperative for drafting management plans that inclusive of self-management education, dietary follow up, physical activity and behavior alteration to optimize blood glucose and diminish diabetes-related complications. The review of the issue is still relatively limited until more studies on this area have been conducted.

## Conclusion

Diabetes is a complicated illness that requires individual patient to adhere to various recommendations in making day-to-day choices in regard to diet, physical movement, and medications. It additionally requires the personal capability of diverse self-management abilities. There is an enormous need for committed self-care practices in various spaces, with nutritional choices, physical activity, legitimate medication, and blood glucose monitoring by the patients. A positive and encouraging self-care exercise commitment for diabetic patient can be emanated from good social support. Parental support in disease management leads to an effective change in patients' glycaemic control. Nevertheless, the majority of adolescent patients with T2DM are associated to families with sedentary daily routines, high-fat diets, and poor food habits who often have a family history of diabetes. This is likely to be disadvantageous to the management of diabetes in adolescents. The responsibility of clinicians in advancing self-care is imperative and ought to be highlighted. To prevent any long-term complications, it is important to recognize the comprehensive nature of the issue. An orderly, multi-faceted and coordinated progress must be involved to advance self-care practices.

## Author contributions

CN, LM, YW, and MS designed and directed the study. They were involved in the planning and supervised the study. JE, YK, CN, LM, YW, MH, YH and MS were involved in the interpretation of the data, as well as provided critical intellectual content in the manuscript. JE contributed to writing the manuscript and updated and revised the manuscript to the final version with the assistance of other authors.

### Conflict of interest statement

The authors declare that the research was conducted in the absence of any commercial or financial relationships that could be construed as a potential conflict of interest.
